# Serological association between *Leishmania infantum* and sand fly fever Sicilian (but not Toscana) virus in sheltered dogs from southern Portugal

**DOI:** 10.1186/s13071-017-2023-x

**Published:** 2017-03-13

**Authors:** Carla Maia, Sulaf Alwassouf, José Manuel Cristóvão, Nazli Ayhan, André Pereira, Remi N. Charrel, Lenea Campino

**Affiliations:** 1 Global Health and Tropical Medicine, GHMT, Instituto de Higiene e Medicina Tropical, IHMT, Universidade Nova de Lisboa, UNL, Rua da Junqueira, 100, 1349-008 Lisboa, Portugal; 2 UMR “Emergence des Pathologies Virales” (EPV: Aix-Marseille University - IRD 190 - Inserm 1207 - EHESP), Marseille, France; 3 Fondation IHU Méditerranée Infection, APHM Public Hospitals of Marseille, Marseille, France

**Keywords:** Dog, *Leishmania infantum*, Phlebovirus, Portugal, Sand fly-borne disease, Sand fly fever Sicilian virus, Serology, Toscana virus

## Abstract

**Background:**

Phlebotomine sand fly-borne diseases such as leishmanioses and phleboviruses are emerging threats to animal and public health. Canine leishmaniosis caused by *Leishmania infantum* is an endemic zoonosis in Portugal. Antibodies to Toscana virus (TOSV) and sand fly fever Sicilian virus (SFSV) were also reported in dogs from the south of the country. The aim of this work was to evaluate a possible association between exposure to *L. infantum*, TOSV and SFSV in sheltered dogs from the south of Portugal.

**Results:**

Seventy-six (13.1%) out of 581 dogs were seropositive for *L. infantum*, 327 (56.3%) for SFSV and 36 (6.2%) for TOSV. Six dogs were co-exposed with *L. infantum* and TOSV, 51 with *L. infantum* and SFSV and 25 with TOSV and SFSV. One dog had antibodies to the three pathogens. *Leishmania infantum* seroprevalence was significantly higher in pure breed dogs than in mongrels and in dogs with clinical signs while SFSV positivity was significantly higher in males, in pure and cross-breed dogs than in mongrels and in those not treated with insecticides. Seroprevalence for both viruses was significantly higher in dogs over than 7 years-old than in those aged 1–7. A significant association was observed between the presence of antibodies to *L. infantum* and SFSV.

**Conclusions:**

The presence of antibodies to several phlebotomine sand fly-borne pathogens in dogs, reinforces the need to implement efficient prophylactic measures to prevent infection among vertebrate hosts including humans. The results also indicate that dogs are good sentinels for assessing human exposure to TOSV and SFSV. Further studies must be performed to elucidate the role of dogs in the dynamics of transmission and if they can play a role as amplifying or reservoir hosts in the natural cycle of these viruses. Public and animal health impacts of these phleboviruses in Portugal should be addressed *via* serological and virological studies on both phlebotomine sand flies and vertebrate hosts, especially on humans.

## Background

Phlebotomine sand flies (Diptera: Psychodidae: Phlebotominae) are the vectors of protozoan parasites of the genus *Leishmania* and of several viruses mostly belonging to the genus *Phlebovirus* of the family *Bunyaviridae* [1–3]. Zoonotic visceral leishmaniosis caused by *L. infantum* is the most dangerous form of *Leishmania* infection, being lethal when untreated. Domestic dogs are the main reservoir hosts for human infection. Canine leishmaniosis (CanL) is endemic in Portugal, with a nation-wide overall seroprevalence of 6.3%, being higher than 12% in some districts [4] while human visceral leishmaniosis is considered a hypoendemic disease [5].

Sand fly-borne phleboviruses pathogenic to humans such as Toscana virus (TOSV), sand fly fever Naples (SFNV) and sand fly fever Sicilian (SFSV) viruses are endemic in the Mediterranean region [1, 2]. Although most of human infections are either asymptomatic or influenza-like syndromes, TOSV has been considered emergent, as outbreaks and sporadic cases of acute meningitis or meningo-encephalitis due to this virus have been reported in southern Europe [1, 6]. On the other hand, SFNV and SFSV also frequently cause epidemics of febrile illness during the summer [1]. In Portugal, human cases of TOSV meningitis have been reported [7–10] along with the detection of antibodies to TOSV in blood donors, professionals with outdoor activity, environmental health care technicians, bird banders, hunters, individuals seropositive to *Leishmania* and individuals with symptoms and laboratory diagnostic request for vector-borne viruses [10, 11]. The presence of SFSV antibodies has also been detected in human sera [12]. In addition, Alcube, Arrabida and Massilia viruses have been isolated from phlebotomine sand flies collected in the southwest of Portugal [13, 14]. Although no animal reservoir hosts of these viruses have been identified yet, recent data showed that domestic animals are frequently exposed to them [15–20]. Seroprevalence rates up to 6.8% for TOSV and up to 50.8% for SFSV were recently described in dogs from Portugal [21].

As phlebotomine sand flies are the common vectors of *Leishmania* and of several phleboviruses, demonstration of an epidemiological link between human leishmaniosis and TOSV infections in southeast France was not unexpected [22]; this finding reinforces the importance of better understanding the interactions among sand fly-borne phleboviruses, *Leishmania* parasites, vectors and vertebrate hosts in order to ascertain their impact on public and animal health. Thus, the aim of the present study was to investigate a possible association between exposure to *Leishmania*, TOSV and SFSV in sheltered dogs from Lisboa and Setúbal districts, south of Portugal, where the presence of the protozoan [23] and both phleboviruses [21] was previously reported.

## Methods

### Animals and samples

In May 2011 and in May 2012, 581 dogs from five shelters from Lisboa and Setúbal districts in the south of Portugal were studied. The reasons for choosing dogs from these districts were: (i) a canine seroprevalence of 5.4–6.6% to TOSV and of 47.7–54.5% to SFSV [21]; (ii) a CanL prevalence ranging from 5.9% in dogs apparently healthy attending veterinarian clinics to 28.6% in stray dogs [4, 24]; (iii) the description of human cases of visceral leishmaniosis [25] and TOSV meningitis [10]; and (iv) the isolation of *L. infantum* [26, 27] and various phleboviruses (Alcube, Arrabida and Massilia) from phlebotomine sand flies [13, 14]. Informed consent was obtained from the legal detainer, i.e. the person in charge of the rescue association. Blood samples (2–3 ml) were collected by cephalic or jugular venipuncture. Serum was separated by centrifugation and stored at -20 °C until use. Whenever available, data on gender, age (distributed according to 3 classes: young, 1–11 months; adult, 12–83 months; senior, ≥ 85 months), breed, use of insecticides, and clinical status, i.e. absence or presence of at least one sign compatible with leishmaniosis, namely muscular atrophy, dermatological manifestations, epistaxis, lameness, lymphadenopathy, onychogryphosis, ocular manifestations, pale mucous or weight loss, were recorded for each dog. Dogs with at least one clinical sign were considered suspected of leishmaniosis. Serum samples were selected from previous epidemiological studies regarding exposure to *L. infantum* [23] or to TOSV and SFSV [21].

### Detection of anti- *Leishmania* IgG

Immunoglobulin G antibodies against *Leishmania* in canine sera were detected using an enzyme-linked immunosorbent assay (ELISA) kit (Bordier Affinity Products SA, Crissier, Switzerland) according to the manufacturer’s guidelines [28]. The result was considered positive when the absorbance of the analyzed sample was higher than the absorbance of the weak positive control serum provided with the kit.

### Virus microneutralisation assay

Each serum was tested using microneutralisation assay in 96-well microtitre plates using Vero cells as previously described [16, 19]. Briefly, 2-fold serial dilutions of 50 μl of sera samples were mixed with an equal volume of 1,000 TCID_50_ of TOSV (strain MRS2010-4319501) or SFSV strain Sabin [29] into 96-well plates, providing 2-fold final dilutions between 1:20 and 1:160. Controls consisted of each serum (1:10) with Vero cells but without virus. After five days of incubation at 37 °C in presence of 5% CO_2_, the microplates were read under an inverted microscope and the presence (neutralization titre at 10, 20, 40, 80 and 160) or absence (no neutralization) of cytopathic effect was noted. Cut-off value for positivity was set at titre ≥ 20 [16, 19].

### Statistical analysis

The exact binomial test established confidence intervals (CI) with a 95% confidence level. The Chi-square, Fisher’s or Fisher-Freeman-Halton exact tests were used to compare percentages of positivity among categories of the same independent variables and also the total prevalence of each agent. Correlation between pathogens was also calculated. A *P-*value < 0.05 was considered as statistically significant. For factors with significant results, odds ratio (OR) was presented. Analyses were performed with SPSS® 23 software for Windows and Epi Info™ 7.1.5.2 software from Centers for Disease Control and Prevention.

## Results

Of the 581 sera, 76 (13.1%) were positive for *Leishmania infantum*, 327 (56.3%) for SFSV and 36 (6.2%) for TOSV (Table 1). Six dogs presented IgG against *L. infantum* and TOSV, 51 against *L. infantum* and SFSV and 25 against TOSV and SFSV. Only one dog had antibodies to the three pathogens. *Leshmania infantum* seroprevalence was significantly higher in pure breed dogs than in mongrels (*P* = 0.016; OR = 2.11, 95% CI: 1.13–3.92) and in dogs with clinical signs (*P* = 0.001; OR = 2.44, 95% CI: 1.45–4.10) while SFSV positivity was significantly higher in males (*P* = 0.005; OR = 1.64, 95% CI: 1.16–2.31), in pure or in mongrels than in cross-breed dogs (*P* = 0.008; OR = 2.25, 95% CI: 1.23–4.11 and *P* = 0.001; OR = 2.12, 95% CI: 1.35–3.32, respectively) and in those not treated with insecticides (*P* = 0.018; OR = 1.64, 95% CI: 1.09–2.48). Seroprevalence rates of SFSV and TOSV were significantly higher in dogs over 7 years old than in those aged 1–7 (*P* < 0.0001; OR = 3.53; 95% CI: 2.28–5.48 and *P* = 0.007; OR = 2.64, 95% CI: 1.27–5.50, respectively). Dogs presenting antibodies to *Leishmania* were significantly more at risk to have antibodies to SFSV (*P* = 0.041; OR = 1.69, 95% CI: 1.02–2.82) (Table 2).

**Table 1:** Seroprevalence and risk factors to *Leishmania infantum*, SFSV and TOSV in sheltered dogs from Portugal

Variable/Category	No. of tested dogs (%)				No. of seropositive dogs (%)		
		*Leishmania infantum*	OR (95% CI)	Sand fly fever Sicilian virus	OR (95% CI)	Toscana virus	OR (95% CI)
Gender	567	*χ* ^2^ = 1.011, *df* = 1, *P* = 0.315	–	*χ* ^2^ = 7.920, *df* = 1, *P* = 0.005	1.636 (1.160–2.307)	*χ* ^2^ = 0.514, *df* = 1, *P* = 0.474	–
Female	340 (60.0)	41 (12.1)		175 (51.5)		23 (6.8)	
Male	227 (40.0)	15.0 (34)		144 (63.4)		12 (5.3)	
Breed	568	*χ* ^2^ = 5.746, *df* = 2, *P* = 0.057	–	*χ* ^2^ = 11.654, *df* = 2, *P* = 0.003		*χ* ^2^ = 0.202, *df* = 2, *P* = 0.964 (ns)	–
Pure breed	79 (13.9)	17 (21.5)^a^		48 (60.8)^b^		4 (5.1)	
Crossed breed	98 (17.3)	13 (13.3)		40 (40.8)^b,c^		6 (6.1)	
Mongrel	391 (68.8)	45 (11.5)^a^		232 (59.3)^c^		25 (6.4)	
Age (months)	526	*P* = 0.244^*^(ns)	–	*P* < 0.0001^*^		*P* = 0.013^*^	
≤ 11	6 (1.1)	0 (0.0)		3 (50.0)		1 (16.7)	
12-83	377 (71.7)	44 (11.7)		183 (48.5)^d^		16 (4.2)^e^	
≥ 84	143 (27.2)	24 (16.8)		110 (76.9)^d^		15 (10.5)^e^	
Insecticides	580	*χ* ^2^ = 0.408, *df* = 1, *P* = 0.523	–	*χ* ^2^ = 5.641, *df* = 1, *P* = 0.018	1.642 (1.088–2.479)	*χ* ^2^ = 0.001, *df* = 1, *P* = 0.974	–
No	466 (80.3)	59 (12.7)		274 (58.8)		29 (6.2)	
Yes	114 (19.7)	17 (14.9)		53 (46.5)		7 (6.1)	
Clinical signs	580	*χ* ^2^ = 11.733, *df* = 1, *P* = 0.001	2.435 (1.446–4.100)	*χ* ^2^ = 0.807, *df* = 1, *P* = 0.369	–	*χ* ^2^ = 3.571, *df* = 1, *P* = 0.059	–
Absent	460 (79.3)	49 (10.7)		255 (55.4)		33 (7.2)	
Present	120 (20.7)	27 (22.5)		72 (60.0)		3 (36)	
Total	581	76 (13.1)		327 (56.3)		36 (6.2)	

*Abbreviation*: *ns* statistically significant difference(s) not confirmed after pairwise comparisons

^a^ *χ* ^2^ = 5.75, *df* = 1, *P* = 0.016, OR = 2.108 (1.134–3.919)

^b^ *χ* ^2^ = 6.959, *df* = 1, *P* = 0.008, OR = 2.245 (1.226–4.112)

^c^ *χ* ^2^ = 10.887, *df* = 1, *P* = 0.001, OR = 2.116 (1.348–3.320)

^d^ *χ* ^2^ = 33.95, *df* = 1, *P* < 0.0001, OR = 3.534 (2.280–5.477)

^e^ *χ* ^2^ = 7.213, *df* = 1, *P* = 0.007, OR = 2.644 (1.271–5.501)

^*^ *P*-value obtained by the Fisher-Freeman-Halton exact test

**Table 2:** Serological association between *Leishmania infantum*, SFSV and TOSV in sheltered dogs from Portugal

Variable/Category	No. of tested dogs (%)	No. of seropositive dogs (%)			
		Sand fly fever Sicilian virus	OR (95% CI)	Toscana virus	OR (95% CI)
*Leishmania infantum*	581	*χ* ^2^ = 4.163, *df* = 1, *P* = 0.041	1.693 (1.017–2.818)	*P* = 0.451*	1.357 (0.545–3.377)
seronegative	505	276 (54.7)		30 (5.9)	
seropositive	76	51 (67.6)		6 (7.9)	

^*^ *P*-value obtained by the Fisher’s exact test

## Discussion

In the context of climate change and globalization, phlebotomine sand fly-borne diseases caused by *Leishmania* protozoan parasites and viral agents, among which the most important are grouped into the genus *Phlebovirus*, are emerging threats to public and animal health worldwide. In fact, in the Mediterranean region the geographical ranges of several agents of these diseases and their frequency are expanding [1, 3, 5, 30, 31]. Some of the assumptions that domestic dogs fulfil as reservoir hosts of *L. infantum* is the close relationship with humans together with the exposure to the bites of the same sand fly vectors [32]. Therefore, a similar presumption can be made regarding the role of dogs as reservoir, amplifying hosts or sentinels for human infections caused by sand fly-borne viruses. Thus, the present study was carried out to investigate a possible association between TOSV, SFSV and *L. infantum* exposure in sheltered dogs and to evaluate the main implications for public and animal health.

The sera samples examined in the present study were not collected to determine seroprevalence values but with the aim to investigate the possible relationship between exposure to the protozoan and to both phleboviruses. However, the percentage of dogs with antibodies to *L. infantum* (13.1%): (i) was higher than the one previously obtained in the same region but in dogs attending veterinarian clinics randomly selected (6%; [4]) or apparently clinically healthy (5.9%; [24]); (ii) was lower than the one obtained in dogs with clinical signs compatible with a canine vector borne disease (27.2%; [24]) or in stray dogs (28.6%; [27]) confirming the endemicity of CanL in Lisboa and Setúbal districts. The seroprevalence to TOSV (6.2%) observed here was in the same order of magnitude as those recently reported in dogs in Algeria (4.3%; [20]), in Cyprus (8.4%; [18]), in France (3.9%; [19]), in Greece (4.4%; [18]) and in Tunisia (6.8%; [16]) reinforcing that dogs can harbour TOSV and their role as sentinels should be investigated. Similarly, the seroprevalence to SFSV (56.3%) obtained in the present study reinforces previous results from Cyprus (60.2%; [18]), Greece (71.9%; [18]) and Tunisia (50.8%; [16]) that dogs are frequently exposed to the virus.

The present data revealed that sheltered dogs are a blood source for phlebotomine sand flies probably due to the lack of regular access to prophylaxis against ectoparasites (in the present study only 19.7% of the dogs were treated with an insecticide). In fact, the risk to possess SFSV neutralising antibodies was 1.6 times higher in dogs not using insecticides in comparison to the ones that use it, which is in agreement with the data obtained by Cortes et al. [4] where dogs not protected against insects had 1.5 times higher risk of *Leishmania* infection. Gender predisposition to *Leishmania* infection has been a field of discussion, with some studies reporting a higher prevalence in male dogs, others in female dogs and others not finding any differences among them [33]. With the exception of the work by Alwassouf et al. [18] in Greece where seroprevalence of SFSV was higher in female dogs, and the present study, where seroprevalence to SFSV was higher in male dogs, no studies of gender predisposition to sand fly-borne phlebovirus have been performed.

On the other hand, the higher seroprevalence to *L. infantum* in pure breed dogs in comparison with mongrels observed in the present study might be related to a certain level of resistance to disease development by the latter [33] as previously observed in a national CanL survey [4]. Whether or not all dog breeds are identically susceptible to SFSV infection is not known, but the rate of SFSV neutralising antibodies was significantly lower in crossed breed dogs compared with pure breed. As increasing of antibody levels has been associated with clinical progression of disease, it is not surprising that we found that antibodies to *L. infantum* were significantly higher in dogs presenting clinical signs compatible with leishmaniosis [34]. Seroprevalence to *L. infantum* (albeit not statistically significant) increased with age probably associated to a cumulative exposure of older animals to the protozoan [33]. Furthermore, the increased contact with the vectors was likely the reason for the age trend observed for TOSV and SFSV antibodies in previous canine surveys [16, 19]. Although in the present study this trend was not observed, the seroprevalence to both viruses was significantly higher in dogs older than 7 years than in those aged 1 to 7 years.

Co-infection with *Leishmania* spp. and different sand fly-borne phleboviruses in the insect on one hand [35, 36] and in dogs on the other hand [17] have been reported. In addition, the detection of *L. infantum* and TOSV and Massilia virus in phlebotomine sand flies [13, 37], humans [22] or dogs [38] sharing the same environmental area has also been described. Thus, we evaluated if the seroprevalence to one of the tested pathogens was significantly associated with seroprevalence to the others. An association between the presence of antibodies to *L. infantum* and SFSV was observed as 51 dogs presented IgG against both microorganisms. Since there is no cross-reactivity between *Leishmania* and SFSV, dogs exposed to the protozoan are at greater risk of being exposed to SFSV (and *vice versa*). The capacity of SFSV to cause disease in dogs is currently unknown, nevertheless it is probably not pathogenic to them as no association between the presence of clinical signs and the exposure to this phlebovirus was observed. However, and since it can cause disease in humans, it will be important to determine if dogs may act as a potential reservoir or amplifying hosts for this pathogen. The only human data regarding the circulation of SFSV in Portugal were obtained in the seventies [12] with a haemagglutination technique prone to cross-react with phleboviruses from other serocomplexes [39]. Therefore, and as our results imply the presence of either SFSV or an SFSV-related virus in Portugal, it would be important to pursue serological studies in humans based on neutralization-assays to determine their level of exposure to SFSV. In addition, attempts to isolate SFSV or another virus genetically related to SFSV from phlebotomine sand flies and vertebrate hosts should be made as no strain has been isolated yet in Portugal.

In the present study no association between the presence of antibodies to *L. infantum* and TOSV was detected (only six dogs were seropositive for both pathogens), not corroborating the epidemiological relationship between human [22] and canine [38] leishmaniosis and TOSV infection previously suggested. Nevertheless, the seroprevalence rate obtained in dogs in the present study, together with the seroprevalence detected in humans (ranging from 2% in control population to 4.2% in population with central nervous system disease) [10] and the reports of human cases of TOSV meningitis [9, 10] demonstrate the circulation of the virus or TOSV-like viruses in Portugal.

Our data demonstrate the co-circulation of *Leishmania* and both phleboviruses in the same region, thus the detection of neutralizing antibodies to both TOSV and SFSV in 25 dogs and the presence of antibodies to the three microorganisms in one dog was not unexpected and is congruent with entomological data regarding the presence of some of the sand fly species responsible or putative responsible for their circulation [1, 3, 36, 39], namely *Phlebotomus ariasi*, *Phlebotomus perniciosus*, *Phlebotomus sergenti* and *Sergentomyia minuta* [40]. Given the low probability of co-infections by different pathogens in phlebotomine sand flies, as observed in nature [35, 36], it can be speculated that the mechanism leading to a double and even a triple serological reactivity in vertebrate hosts is more likely to occur due to successive bites by phlebotomine sand flies, of the same or of different species, infected by a single pathogen [17, 22].

## Conclusions

This study demonstrates the exposure to and co-circulation of phlebotomine sand fly-borne phleboviruses belonging to two distinct genetic and antigenic groups and *Leishmania infantum* in sheltered dogs, reinforcing the need to implement efficient prophylactic measures to prevent sand fly-borne infections among vertebrate hosts including humans. The results also indicate that dogs are good sentinels for assessing human exposure to TOSV and SFSV and since both viruses are proven human pathogens, further studies must be performed to elucidate if dogs play a role as reservoir or amplifying hosts in the natural cycle of these viruses. It is also a matter of priority to address the public and animal health impacts of these phleboviruses in Portugal via serological and virological studies on both phlebotomine sand flies and vertebrate hosts, especially on humans.

## Abbreviations

CanL: Canine leishmaniosis
CI: Confidence intervals
ELISA: Enzyme-linked immunosorbent assay
OR: Odds ratio
SFNV: Sand fly fever Naples virus
SFSV: Sand fly fever Sicilian virus
TOSV: Toscana virus
